# Study of the effects of oral zinc supplementation on peroxynitrite levels, arginase activity and NO synthase activity in seminal plasma of Iraqi asthenospermic patients

**DOI:** 10.1186/1477-7827-12-1

**Published:** 2014-01-03

**Authors:** Mahmoud Hussein Hadwan, Lamia A Almashhedy, Abdul Razzaq S Alsalman

**Affiliations:** 1 Chemistry Department, College of Science, Babylon University, Babylon, Iraq; 2 Surgery Department, College of Medicine, Babylon University, Babylon, Iraq

**Keywords:** Asthenospermia, Nitric oxide synthase, Arginase, Peroxynitrite, Zinc, Zinc supplementation, Oxidative stress

## Abstract

**Background:**

Low concentrations of nitric oxide (NO) are necessary for the biology and physiology of spermatozoa, but high levels of NO are toxic and have negative effects on sperm functions. Although several studies have considered the relationship between infertility and semen NO concentrations, no study on the effects of asthenospermia treatments such as oral zinc supplementation on concentrations of NO, which are important in fertility, has been reported. Studies have shown that oral zinc supplementation develops sperm count, motility and the physical characteristics of sperm in animals and in some groups of infertile men. The present study was conducted to study the effect of zinc supplementation on the quantitative and qualitative characteristics of semen, along with enzymes of the NO pathway in the seminal plasma of asthenospermic patients.

**Methods:**

Semen samples were obtained from 60 fertile and 60 asthenozoospermic infertile men of matched age. The subfertile group was treated with zinc sulfate; each participant took two capsules (220 mg per capsule) per day for 3 months. Semen samples were obtained (before and after zinc sulfate supplementation). After liquefaction of the seminal fluid at room temperature, routine semen analyses were performed. The stable metabolites of NO (nitrite) in seminal plasma were measured by nitrophenol assay. Arginase activity and NO synthase activity were measured spectrophotometrically.

**Results:**

Peroxynitrite levels, arginase activity, NO synthase activity and various sperm parameters were compared among fertile controls and infertile patients (before and after treatment with zinc sulfate). Peroxynitrite levels and NO synthase activity were significantly higher in the infertile patients compared to the fertile group. Conversely, arginase activity was significantly higher in the fertile group than the infertile patients. Peroxynitrite levels, arginase activity and NO synthase activity of the infertile patient were restored to normal values after treatment with zinc sulfate. Volume of semen, progressive sperm motility percentage and total normal sperm count were increased after zinc supplementation.

**Conclusions:**

Treatment of asthenospermic patients with zinc supplementation leads to restored peroxynitrite levels, arginase activity and NO synthase activity to normal values and gives a statistically significant improvement of semen parameters compared with controls.

**Trial registration:**

ClinicalTrials.gov identifier: NCT01684059

## Background

Infertility is defined as the lack of ability to conceive within 1 year of unprotected intercourse with the same partner [1]. It is estimated that nearly 13–15% of couples are infertile [2]. As a result of this prevalence, couples proceed to recognize either partner’s responsibility. Half of these infertile couples involve a component of male infertility [3]. Male subfertility commonly comprises a status in which the incapability to conceive is related to an alteration present in the male partner [4].

There are several causes leading to male infertility, such as oxidative stress or nutritional insufficiency of trace elements, i.e., selenium and zinc (Zn) [5,6]. The trace element Zn is second only to iron as the most abundant element in the human tissues. Although Zn is found in most types of foods such as red meat, white meat, fish, and milk, the World Health Organization (WHO) estimates that one-third of the world population is deficient in Zn [7]. Zinc serves as a cofactor for more than 80 metallic enzymes concerned in cellular development processes such as DNA transcription and protein synthesis; for this reason, Zn is expected to be important for reproduction. Zinc is essential for testicular steroidogenesis, testicular development, synthesis and secretion of luteinizing hormone and follicle stimulating hormone, testosterone synthesis, gonadal differentiation, formation and maturation of spermatozoa, acrosome reaction, acrosin activity, and fertilization [8,9]. Also, Zn has anti-apoptotic [10] and antioxidant properties [11]. Ozturk *et al*. [12] have indicated that Zn deficiency in rats results in atrophy of seminiferous tubules and interruption of spermatogenesis. Lewis-Jones *et al*. [13] pointed to the involvement of Zn in functions that are important for sperm physiology such as sperm membrane integrity, increased sperm motility and regulation of the spiral movements of the sperm tail.

O_2_ is required to support the life of spermatozoa, but its metabolites, such as reactive oxygen species (ROS), have some disadvantages, which include modification of cellular functions and/or threaten cell survival [14]. For this reason, ROS formation must be regulated to sustain only the necessary amount that suitable for maintaining normal cellular function.

A sequence of enzymatic and non-enzymatic antioxidants normally protects the spermatozoa against oxidants [15]. A major source of the antioxidant enzymes in the ejaculate is the male accessory sex gland secretions, which have been shown to be responsible for preserving sperm DNA integrity from oxidative stress experienced in the uterine environment [16].

Nitric oxide (NO) is a free radical that is formed by most tissues of the human body and participates in a wide range of biological processes [17]. NO is synthesized through the enzymatic conversion of L-arginine to L-citrulline by the action of one of the isoenzymes known as nitric oxide synthase (NOS), and is concerned with diverse physiological functions in various organs, including the human male reproductive tracts [18,19].

Several studies have been done to recognize the function of NO in the male reproductive tract after discovery of a unique isoform of NOS as testis-specific nNOS (TnNOS) [20]. A specific conclusion from these studies suggests that NO in low levels is necessary to complete a group of male reproductive functions such as spermatogenesis, spermiogenesis, sperm motion, acrosome reaction, sperm/oocyte fusion and sperm capacitation [21-23]. On the other hand, high levels of NO have injurious effects on sperm properties such as motility, morphology and DNA stability [24,25]. Also, high levels of NO act to increase apoptosis and lipid peroxidation [26,27].

Given the key role of oxidative stress in the pathogenesis of male subfertility, treatment strategies with the purpose of reducing levels of seminal oxidative stress are required for the assisted reproduction outcome and natural pregnancy. Many antioxidant treatments have been used in the hope of developing sperm quality. A wide range of therapies have been used over the years consisting of many different vitamins, such as vitamins A, E and C, and compounds, including carnitines, phosphatidylcholine, kallikrein and pentoxifylline, without particular interest to neutralize the lipoperoxidative injury [28].

Studies have shown that oral Zn supplementation develops sperm count, motility and the physical characteristics of sperm in animals [29,30], and in some groups of infertile men [31-33]. The present study was conducted to examine the effects of Zn supplementation on the quantitative and qualitative characteristics of semen, along with peroxynitrite levels, arginase activity and NO synthase activity in the seminal plasma of asthenozoospermic patients.

## Methods

### Ethical committee

Iraq: Ethics Committee (University of Babylon/ College of Science), Reference number of approval: 545 Date: 22/6/ 2011.

### Patients

This study included 60 subfertile male partners from couples who had consulted the infertility clinic of the Babil Hospital of Maternity (Hilla City, Iraq) from July 2011 to July 2012. The approval of the institutional research ethics committee and signed written consent of every patient included in the study was obtained. A detailed medical history was taken and physical examination was performed. Subjects currently on any medication or antioxidant supplementation were not included. The inclusion criteria were asthenozoospermia, the absence of endocrinopathy, varicocele and female partner infertility. Smokers and alcoholic men were excluded from the study because of their recognized high seminal ROS levels and decreased antioxidant levels. The selection criteria of the fertile group were the absence of asthenozoospermia, endocrinopathy, varicocele and having a birth in the last year. Semen samples were obtained (before and after Zn sulfate supplementation). After liquefaction of the seminal fluid at room temperature, routine semen analyses including semen volume, pH, concentration, sperm motility, normal sperm morphology and round cells were performed according to the 2010 WHO recommendation [34].

### Preparation of seminal plasma and spermatozoa for biochemical analysis

For each sample, seminal plasma was separated from the spermatozoa 1 h after semen collection by centrifugation of 2 mL of seminal fluid at 1500 × *g* for 10 min at 4°C and maintained at -30°C until analysis. The pellet was resuspended in 10 volumes of NTPC medium [113 mM NaCl (0.66 g/100 mL), 2.5 mM NaH_2_PO4 (0.3 g/100 mL), 2.5 mM Na_2_HPO4 (0.0355 g/100 mL), 1.7 mM CaCl_2_ (0.0188 g/100 mL), 1.5 mM D-glucose (0.0027 g/100 mL), 20 mM Tris (0.242 g/100 mL), 0.4 mM EDTA (0.0148 g/100 mL) adjusted to pH 7.4 with HCl] and centrifuged at 1500 × *g* for 10 min at 4°C. This washing procedure was repeated three times. Triton X-100 (0.1%) was added to the pellets obtained and the samples centrifuged again at 4000 Xg for half an hour in a refrigerated centrifuge. This concentration of Triton X-100 does not affect enzyme levels. The supernatant was used for measurements in spermatozoa.

The samples were classified into three groups called group I (G1, healthy donors), group II (G2, patients before treatment) and group III (G3, patients after treatment). After that, the samples were frozen (-20°C) until analyzed.

### Chemicals

All reagents and chemicals were of analytical grade and obtained from standard commercial suppliers.

### Biochemical procedures

Nitric oxide synthase: The assay for NOS is based on the quantitative conversion of oxyhemoglobin to methemoglobin by NO, which can be followed spectrophotometrically as a decrease in absorbance [35].

Arginase activity: Arginase activity was then determined by the spectrophotometric method described by Geyer and Dabich [36]. One unit of arginase activity is defined as one micromole of urea released per minute at 37°C.

Peroxynitrite determination: The sample containing peroxynitrite was added to phenol in 50 mM sodium phosphate buffer (pH 7.4) mediated nitration of phenol, after incubation for 2 h at 37°C; NaOH was added to produce the salt nitrophenol, which has a maximum absorbance at 412 nm [37]. The yield of nitrophenol was calculated from ϵ 4400 M^-1^ cm^-1^ as an index of peroxynitrite concentrations.

### Statistical analysis

Data analysis was performed using SPSS 16 for Windows (SPSS Inc., Chicago, IL, USA). Data were expressed as mean, SD, SE and range, were evaluated by one-way analysis of variance (ANOVA). The Kolmogorov-Smirnov test was used to verify if data followed normal distribution. Correlations between numeric data were measured with Pearson’s correlation of coefficient. Statistical significance level was considered at P < 0.05.

## Results

The results in Table 1 indicate the baseline characteristics of the semen parameters depicted in the fertile (G1) and subfertile groups (before [G2] and after treatment [G3] with Zn sulfate). These parameters significantly decreased in the infertile group [G2] compared with the healthy donor group [G1] (*P* < 0.05). However, the level of the semen parameters significantly increased (returned to normal values) after zinc sulfate supplementation [G3] compared with subfertile group before treatment [G2] (*P* < 0.05).

**Table 1:** Ejaculate parameters of seminal fluids of infertile patients (n = 60) and healthy donor (n = 60) (Mean ± SD)

	**Volume (mL)**	**Sperm count (×10**^**6**^**)**	**Progressive sperm motility (%)**	**Normal sperm form (%)**
*Healthy donors G1*	2.8 ± 0.53	77 ± 9	69 ± 8	38 ± 9
*Patients before treatment G2*	1.83 ± 0.66*	47 ± 21*	21 ± 9*	21 ± 11
	(P-value = 0.042)	(P-value = 0.023)	(P-value = 0.00)	(P-value = 0.052)
*Patients after treatment G3*	2.39 ± 0.9**	70 ± 15	39 ± 14**	33 ± 7**
	(P-value = 0.037)	(P-value = 0.03)	(P-value = 0.05)	(P-value = 0.041)

Sn1*: significance versus group I (Healthy donors).

Sn2**: significance versus group II (Patients before treatment).

The results of the present study (Tables 2, 3, 4, 5, 6, 7) show a decrease in arginase activity, an increase in NOS activity and an elevation in peroxynitrite levels in spermatozoa and seminal plasma of patients (G2) compared with the healthy group (G1). This difference was statistically significant (*P* < 0.05). However, Zn supplementation restored to normal ranges the arginase activity, NOS activity and elevation of peroxynitrite levels in spermatozoa and seminal plasma in the subfertile men (G3). (Table 8) shows correlation analyses between progressive motility with peroxynitrite levels, arginase activity and NO synthase activity.

**Table 2:** NOS activity (nM/mg protein/hr) in seminal plasma of infertile and healthy donor groups

	**Mean**	**Std. deviation**	**Std. error**	**95% Confidence interval**		**Compared groups**		**P-value**
				**Lower bound**	**Upper bound**			
*G1*	10.01	2.94	0.38	8.44	12.58	1	2	0.014*
							3	0.818
*G2*	28.49	11.25	1.45	8.83	48.15	2	1	0.014*
							3	0.037*
*G3*	9.93	2.87	0.37	7.15	13.70	3	1	0.818
							2	0.037*

**Table 3:** **NOS activity (nM/10**^**8 **^**Spermatozoa/hr) in spermatozoa of infertile and healthy donor groups**

	**Mean**	**Std. deviation**	**Std. error**	**95% Confidence interval**		**Compared groups**		**P-value**
				**Lower bound**	**Upper bound**			
*G1*	30.10	14.49	1.87	21.73	38.46	1	2	0.007*
							3	0.549
*G2*	57.36	14.65	1.89	33.89	80.83	2	1	0.007*
							3	0.028*
*G3*	35.80	10.65	1.37	29.90	41.70	3	1	0.549
							2	0.028*

**Table 4:** Arginase activity (U/L) in seminal plasma of infertile and healthy donor groups

	**Mean**	**Std. deviation**	**Std. error**	**95% Confidence interval**		**Compared groups**		**p-value**
				**Lower bound**	**Upper bound**			
*G1*	149	49.56	6.39	112.55	187.36	1	2	0.022*
							3	0.713
*G2*	106	37.06	4.78	82.05	130.24	2	1	0.022*
							3	0.05*
*G3*	143	23.52	3.03	133.12	152.99	3	1	0.713
							2	0.05*

**Table 5:** **Arginase activity (U/10**^**8 **^**spermatozoa) in spermatozoa of infertile and healthy donor groups**

	**Mean**	**Std. deviation**	**Std. error**	**95% Confidence interval**		**Compared groups**		**P-value**
				**Lower bound**	**Upper bound**			
*G1*	31.25	6.30	0.81	24.80	37.70	1	2	0.023*
							3	0.820
*G2*	18.98	7.15	0.92	11.73	26.22	2	1	0.023*
							3	0.015*
*G3*	32.45	7.50	0.96	22.94	41.95	3	1	0.820
							2	0.015*

**Table 6:** Peroxynitrite concentrations (μmol/L) in seminal plasma of infertile and healthy donor groups

	**Mean**	**Std. deviation**	**Std. error**	**95% Confidence interval**		**Compared groups**		**P-value**
				**Lower bound**	**Upper bound**			
*G1*	33.63	13.14	1.69	27.09	40.17	1	2	0.030*
							3	0.912
*G2*	44.29	19.43	2.5	34.62	53.95	2	1	0.030*
							3	0.023*
*G3*	33.1	7.94	7.94	29.15	37.05	3	1	0.912
							2	0.023*

**Table 7:** **Peroxynitrite concentrations (μmol/ 10**^**8 **^**spermatozoa) in spermatozoa of infertile and healthy donor groups**

	**Mean**	**Std. deviation**	**Std. error**	**95% Confidence interval**		**Compared groups**		**P-value**
				**Lower bound**	**Upper bound**			
*G1*	18.34	6.43	0.83	12.66	20.01	1	2	0.012*
							3	0.970
*G2*	49.76	15.81	2.04	42.00	77.52	2	1	0.012*
							3	0.014*
*G3*	20.77	6.33	0.81	15.14	26.41	3	1	0.970
							2	0.014*

*The mean difference is significant at the 0.05 level.

**Table 8:** Correlation analyses between progressive motility with peroxynitrite levels, arginase activity and NO synthase activity

	**Progressive motility**			
	**Seminal plasma**		**Spermatozoa**	
	**p-value**	**r-value**	**p-value**	**r-value**
*NOS activity*	0.008	-0.82	0.009	-0.85
*Arginase activity*	0.043	0.89	0.009	0.87
*Peroxynitrite concentrations*	0.041	-0.78	0.008	-0.83

## Discussion

**Figure 1:**
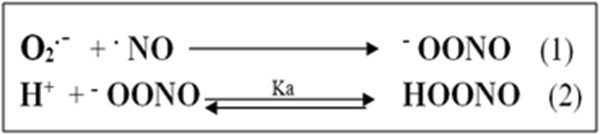
Formation of peroxynitrite ion (Equation 1) and its conjugate acid, peroxynitrous acid (Equation 2).

NO is documented as a novel moderator of sperm function [38] through the modulation of sexual and reproductive functions in mammals [19]. The production of NO in seminal plasma through the action of male reproductive organs or macrophages has been established [19,20]. NO itself is a weak oxidant. However, NO can be altered to an effective oxidant by reaction with superoxide to produce the peroxynitrite ion (^–^OO-N = O) and its conjugate acid, peroxynitrous acid (HOO-N = O) [39], as shown in Figure 1.

Peroxynitrite can react under physiological conditions with electron-rich compounds, such as thiols [40], iron-sulfur centers [41] and zinc-thiolates (metallothioneins) [42]. However, peroxynitrite has a strong one or two electron oxidant and acts as a nitrating agent [43]. It has unexpected stability in solutions because of its strongly oxidizing potential and that it is 36 kcal/mol higher in energy than its isomer nitrate. Folding into a cis-conformation stabilizes peroxynitrite where the negative charge is localized over the entire molecule [44]. In addition, the formation of strong hydrogen bonds with two or three water molecules increased its stability. The restricted reactivity of peroxynitrite with most biological molecules makes it unusually selective as an oxidant, which enhances its influence over biological pathways [44].

In the present study, peroxynitrite levels were higher in seminal plasma of patients with asthenozoospermia, and were negatively associated with concentrations and rapid progressive motility of spermatozoa, as shown in Table 8. These results confirm previous studies [45-47], which suggest that higher NO levels in seminal plasma are correlated with poor sperm quality. Chung *et al*. [48] suggest that NO inhibits sperm motility via the formation of peroxynitrite. This inhibition depends on the production of superoxide from human semen because peroxynitrite is generated by the interaction of NO and superoxide.

The normal function of NO includes neutralized free radicals to avoid the reduction of sperm motility mediated by ROS. However, extreme formation of NO under stress conditions may be harmful and could inhibit sperm motility. Sperm motility is sustained by high concentrations of adenosine triphosphate. It is known that NO acts to decline adenosine triphosphate levels in cells by reduction in the glycolysis pathway and the electron-transport chain [49].

**Figure 2:**
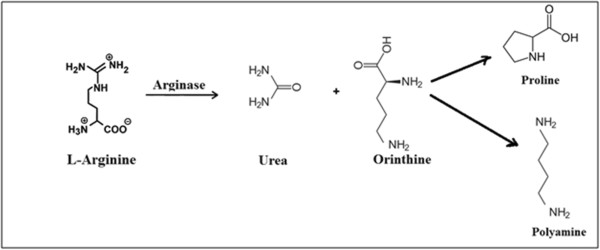
Biosynthetic pathway for production of polyamine.

The rate of NO production may be critically dependent on the availability of arginine, with elevated arginine availability potentially leading to increase NO synthesis. Under physiological conditions, although the K_m_ of the NOS isoforms for arginine is much greater than that of arginase, mammalian arginase competes with NO synthase for the common substrate L-arginine [50], to form urea and L-ornithine. Consequently, arginase can regulate cellular NO synthesis and counteract the biological effects of NO [51,52]. High activity of the arginase enzyme is very important for improving semen quality because it leads to increased polyamines concentrations via consume arginine (Figure 2).

Polyamines are ubiquitous organic polycationic compounds typically included in cell replication and differentiation. These compounds are synthesized from L-ornithine, a nonstandard amino acid involved in the urea cycle by the catalytic activity of ornithine decarboxylase, the rate-limiting enzyme in the biosynthesis of polyamines [53]. Polyamines have been found in the spermatozoa, seminal plasma [54] and epididymis [55], in addition to other tissues of the male reproductive system [56].

Srivastava *et al*. [57] demonstrated that L-arginine supplementation in the culture medium inhibits seminal lipid peroxidation. The probability exists that spermatozoa perform this consequence by synthesizing polyamines, which would need arginase activity, because polyamines protect several tissues against lipid peroxidation [58]. Polyamines exert their cellular effects through the ability to bind nucleic acids and proteins and have been demonstrated to reduce an apoptotic state in human endometrial RL95-2 cells [59]. In addition, L-arginine itself acts as valuable factor for polyamines in causing enhancement of sperm cell motility [54,60]. The complete inhibition of arginase also prevents polyamine biosynthesis in other tissues [61].

**Figure 3:**
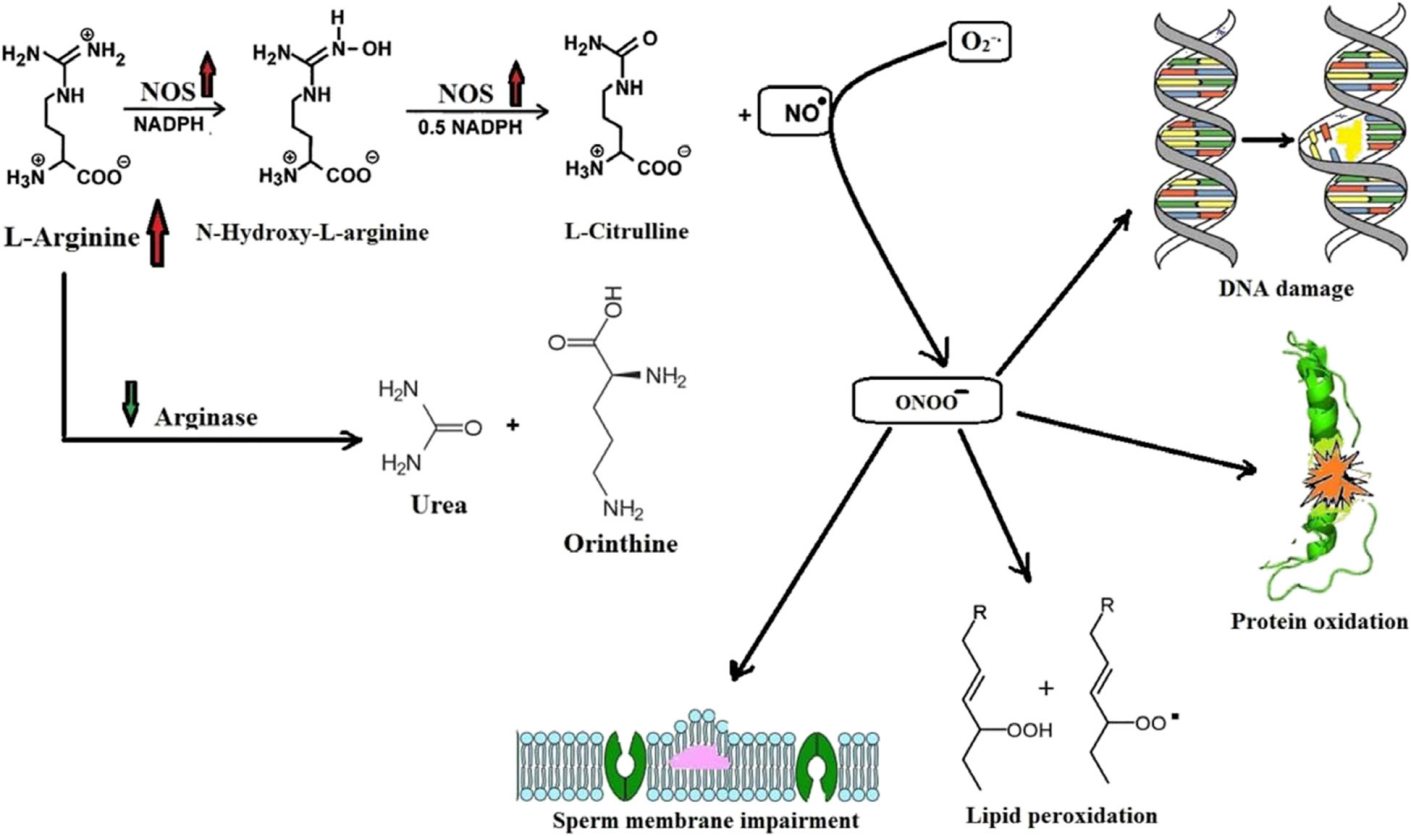
Suggested pathological roles of NO include lipid peroxidation, DNA damage, protein oxidation, and membrane impairment.

The results of the present study (Tables 2, 3, 4, 5, 6) show a decrease in arginase activity, an increase in NOS activity and elevation of peroxynitrite levels. The study suggests that elevated arginine levels could be beyond the lowering of arginase activity. This leads to a relative accumulation of peroxynitrite, the main toxic anion of peroxidation formed from NO. Thus, the high NOS activity could be illustrated directly by the high arginine content found in the semen of patients with asthenozoospermia, given that arginine is a substrate of this enzyme. Therefore, high arginine content necessitates high NOS activity, which may produce increased oxidative stress inclination, as shown in Figure 3.

ROS generation and oxidative status after treatment of asthenozoospermia with zinc sulphate has been studied in detail in our previous studies [33,62] for same patients. These studies were conducted to study the effect of zinc supplementation on the quantitative and qualitative characteristics of semen along with total antioxidant capacity, antiperoxidant activity and lipid peroxidation in seminal plasma of asthenospermic patients. Semen samples were obtained from fertile and asthenozoospermic infertile men with matching age. The subfertile group was treated with zinc sulfate. Total antioxidant capacity and antiperoxidant activity of fertile controls was significantly higher than that of the infertile patient group. These parameters were significantly elevated in the infertile group which treated with zinc sulfate. Lipid peroxidation has opposite behavior.

Zinc supplementation raises arginase activity in the seminal plasma of asthenozoospermic patients to normal values, potentially because of its improvement in the synthesis of metallothioneins (low molecular weight Zn binding protein) that have antioxidant properties [63]. The suitable mechanism of the implication of metallothioneins in the enhancing the quality of seminal fluids was illustrated in previous study [32], which used gel filtration of seminal plasma on Sephadex G-75 for determination the amount of zinc binding proteins of fertile and asthenozoospermic infertile men. The subfertile group consists of the patients which treated with zinc sulfate. The results of the study indicated that zinc supplementation improves the synthesis of zinc binding proteins such as metallothioneins in seminal plasma of asthenozoospermic subjects [33]. Metallothioneins protect biological tissues from damage of oxidative stress via capture harmful oxidant species like the superoxide and hydroxyl radicals [64].

**Figure 4:**
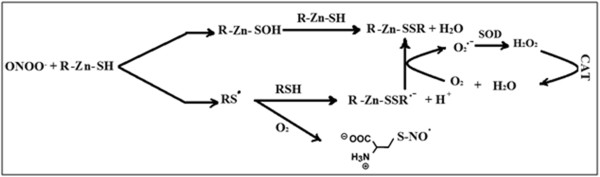
Detoxification of peroxynitrite by thiol and antioxidants enzymes.

Hrabak *et al*. [65] have documented that arginase is inhibited by nitrite, a stable end product of NO. Peroxynitrite will preferably react with reduced thiols (RSH) such as metallothioneins (R-Zn-SH) to produce thiol radicals and sulfenic acid derivatives [66]. These compounds are active and react rapidly with RSH to produce oxidized thiol. Figure 4 elucidates the detoxification of peroxynitrite via R-Zn-SH that are synthesized after zinc supplementation (modified from Calabrese *et al*. [67]).

Thus, the obtainable information supports the fact that although high NO levels are injurious to sperm, low and controlled levels of NO are essential in optimizing specific pathways in sperm physiology.

## Conclusions

Zinc supplementation restores arginase and NOS activity and elevates peroxynitrite levels in spermatozoa and seminal plasma of asthenozoospermic subjects to normal ranges. Zinc supplementation enhances the quantitative and qualitative properties of semen.

## Abbreviations

NO: Nitric oxide; NOS: Nitric oxide synthase; RSH: Reduced thiol; R-Zn-SH: Metallothioneins.

## Competing interests

The authors declare that they have no competing interests.

## Authors’ contributions

All the authors made important roles to the design and viewing of the study. Principally, MHH wrote the manuscript, contributed to the investigation and elucidation of the data. LAA participated in its design and coordination and assisted to draft the manuscript. ARA contributed to the implementation of the protocol. All the authors have been involved in drafting and revising the manuscript, have read, and approved the final manuscript.
